# Origins and pathogenesis of Middle East respiratory syndrome-associated coronavirus: recent advances

**DOI:** 10.12688/f1000research.11827.1

**Published:** 2017-09-01

**Authors:** Stephen A. Goldstein, Susan R. Weiss

**Affiliations:** 1 Department of Microbiology, Perelman School of Medicine, University of Pennsylvania, Philadelphia, PA, USA

**Keywords:** MERS, CoV, origin, pathogenesis

## Abstract

Middle East respiratory syndrome-associated coronavirus (MERS-CoV) has been a significant research focus since its discovery in 2012. Since 2012, 2,040 cases and 712 deaths have been recorded (as of August 11, 2017), representing a strikingly high case fatality rate of 36%. Over the last several years, MERS-CoV research has progressed in several parallel and complementary directions. This review will focus on three particular areas: the origins and evolution of MERS-CoV, the challenges and achievements in the development of MERS-CoV animal models, and our understanding of how novel proteins unique to MERS-CoV counter the host immune response. The origins of MERS-CoV, likely in African bats, are increasingly clear, although important questions remain about the establishment of dromedary camels as a reservoir seeding human outbreaks. Likewise, there have been important advances in the development of animal models, and both non-human primate and mouse models that seem to recapitulate human disease are now available. How MERS-CoV evades and inhibits the host innate immune response remains less clear. Although several studies have identified MERS-CoV proteins as innate immune antagonists, little of this work has been conducted using live virus under conditions of actual infection, but rather with ectopically expressed proteins. Accordingly, considerable space remains for major contributions to understanding unique ways in which MERS-CoV interacts with and modulates the host response. Collectively, these areas have seen significant advances over the last several years but continue to offer exciting opportunities for discovery.

## Introduction

Middle East respiratory syndrome associated-coronavirus (MERS-CoV) was first isolated from a patient with severe, fatal pneumonia in Saudi Arabia in September 2012 ^1^ and was retrospectively identified in Jordan in April 2012 ^2^. To date, the vast majority of the 2,040 confirmed cases and 712 deaths (as of August 11, 2017) have occurred in the Kingdom of Saudi Arabia (World Health Organization), and one large travel-associated outbreak occurred in South Korea ^3^. MERS-CoV has a large (about 30 kb) positive-sense RNA genome characteristic of coronaviruses, encoding conserved replicase and structural genes, and lineage-specific accessory genes are found in the 3′ 10 kb of the genome ^4^.

MERS-CoV research has branched out in several parallel directions. Given its unusually high case fatality rate (about 36%), a paramount concern has been to understand the ecology and emergence of MERS-CoV in order to assess its pandemic potential. Equally important in the interests of developing vaccines and therapeutics is understanding how MERS-CoV actually causes disease, and this has driven extensive work on developing large and small animal models as well as studies of the molecular virus-host interactions that contribute to viral replication and virulence.

This review will examine several of these areas to assess the state of the field as of mid-2017. Specifically, we will focus on four areas: (1) the emerging clarity on the zoonotic origin and evolution of MERS-CoV in bats and camels, (2) development of non-human primate models, (3) generation of transgenic mouse models for studies of pathogenesis and testing of vaccines and therapeutics, and (4) studies attempting to elucidate mechanisms by which MERS-CoV evades or counteracts the host innate immune response. Though not exhaustive, discussion of these areas will provide a clear picture of the state of knowledge in the field and where important gaps remain.

Outstanding questions remain, particularly considering the source of MERS-CoV infections in humans. Although a large percentage of cases report contact with camels, many do not. Large-scale serosurveys suggest rare but widespread subclinical infection ^5^, but it is unknown whether asymptomatically infected individuals can transmit the virus. Notably, seropositive rates among dromedary camels exceeding 90% have been detected in several sub-Saharan African countries in addition to the Middle East, but not a single active MERS-CoV infection has been identified in sub-Saharan Africa. In 2016, sampling of more than 1,000 individuals in Kenya identified two seropositive individuals ^6^, although most of the individuals tested, including the two who tested positive, had little contact with camels. It is unclear whether the absence of cases reported in Africa is due to under-reporting or different ecology of the virus, but further, intensified sampling may fill these gaps.

## Origin and evolution of MERS-CoV

Since the discovery of MERS-CoV, identifying the source of human infections has been considered essential to interrupting zoonotic transmission. Almost immediately, suspicion fell on bats as a likely reservoir. MERS-CoV was classified genomically as a lineage C *betacoronavirus*, a relatively novel lineage typified by the bat coronaviruses HKU4 and HKU5, complete sequences of which had been recovered from bats of the species *Tylonycteris pachypus* and *Pipistrellus abramus* in China, respectively, in 2007 ^7^. Extensive global surveys since the discovery of MERS-CoV have revealed a remarkably wide distribution of lineage C betacoronaviruses in bats, and lineage C betacoronaviruses have since been identified in bats in Italy ^8, 9^, Mexico ^10, 11^, and Thailand ^12^. Most recently, a 2017 global study by Anthony *et al*. ^13^ found that 91 of the 100 phylogenetic coronavirus lineages identified in diverse mammalian orders were found in bats, suggesting that bats are a source of not only lineage C betacoronaviruses but possibly the vast majority of global coronavirus diversity.

In 2013, Memish *et al*. ^14^ surveyed bats in the vicinity of a small human outbreak in Saudi Arabia and reported the identification of a 190-nucleotide (nt) fragment with 100% identity to the MERS-CoV polymerase in a single bat, providing limited evidence that MERS-CoV or a closely related virus circulates in the Arabian Peninsula. However, such a virus could not be isolated from other local or regional bats then or later. Other work appears to be closing in on an origin in sub-Saharan Africa for MERS-CoV.

Also in 2013, Ithete *et al*. ^15^ described a short, 816-nt fragment of coronavirus RNA isolated from a *Neoromicia zuluensis* bat in South Africa that differed by only one amino acid from the equivalent MERS-CoV fragment, a much closer relationship than between MERS-CoV and any other previously described virus ^16^. Analysis of the complete genome sequence of this virus (NeoCoV) by Corman *et al*. ^17^ revealed that it shares 85% nt identity with MERS-CoV across the entire genome and more than 90% amino acid identity, placing the two viruses within the same species. Most recently, a second virus (PREDICT/PDF-2180) in this species was described by Anthony *et al*. ^18^, further supporting the idea that MERS-CoV is descended from an ancestral virus of African bats. Despite the close similarity and conspecific classification of NeoCoV and PREDICT/PDF-2180 with MERS-CoV, the two bat viruses are highly divergent from MERS-CoV in the S1 subunit of the Spike glycoprotein (less than 45% nt amino acid identity), but highly similar to each other (91%), with evidence of recombination between the S1 and S2 subunits, and PREDICT/PDF-2180 was unable to infect human cells. This is consistent with the idea that MERS-CoV has a common ancestor with these viruses but itself arose through the acquisition of a new Spike S1 subunit conferring the ability to infect human cells through its receptor, DPP4 ^18^.

Although a bat origin seems likely, there is no epidemiological link between human MERS-CoV infections and bats, but the epidemiological, genetic, and phenotypic links between dromedary camels and human infection seem conclusive ^5, 19– 24^. Serological evidence of MERS-CoV infection in dromedary camels dates back to at least 1983. In 2014, Müller *et al*. ^25^ reported 81% seropositivity for MERS-CoV in banked dromedary serum samples obtained between 1983 and 1997 in Somalia, Sudan, and Egypt, the first two countries being major exporters of dromedary camels to the Arabian Peninsula. This supports extensive circulation of MERS-CoV in dromedary camels long predating known human cases. Corman *et al*. ^26^ reported similarly high seropositive rates in Kenyan camel serum banked in 1992, and contemporary serum collection shows that high percentages of dromedary camels are also seropositive for MERS-CoV in Nigeria, Tunisia, and Ethiopia ^27^ as well as Burkina Faso and Morocco ^28^. Phylogenetic analysis of MERS-CoV sequences suggests an evolutionary history of MERS-CoV in camels. Sabir *et al*. ^29^ isolated complete MERS-CoV sequences representing five genetic lineages from Saudi Arabian camels. These lineages, including one recombinant lineage that spawned a human outbreak, appeared ancestral to human isolates.

## MERS pathogenesis: insights from humans and non-human primates

Despite intensive research over the last five years, remarkably little is known about MERS-CoV pathogenesis. Owing to religious restrictions on autopsies in MERS-CoV endemic regions, only one post-mortem report has been published ^30^, and no autopsy reports have emerged from outbreaks elsewhere. In the absence of robust post-mortem data from humans, numerous attempts have been made to establish non-human primate models that recapitulate severe human disease caused by MERS-CoV. Although these attempts have been only partially successful, work to this point has illuminated the cellular tropism of MERS-CoV *in vivo* and shed some light on the types of damage and inflammatory responses it causes and elicits in the airway.

The lone autopsy report, stemming from an April 2014 case in the United Arab Emirates ^30^, identifies type 2 alveolar pneumocytes and respiratory multinucleated syncytial cells of uncertain origin as the primary targets of MERS-CoV. Consistent with tropism for cells in the lower airway, the primary pathology observed was diffuse alveolar damage, and there was evidence for immune-mediated pathology in uninfected areas of the lung. No evidence of systemic dissemination of MERS-CoV was found, but data from a single patient cannot rule out the possibility of spread beyond the airway. Animal models are inconsistent on the question of whether MERS-CoV causes systemic infection, and renal failure is a known complication in severe human cases.

### Rhesus macaque model

The earliest MERS-CoV animal model established used rhesus macaques. In 2013 and 2014, de Wit *et al*. ^31^ and Yao *et al*. ^32^, respectively, reported that rhesus macaques infected with MERS-CoV experience self-limiting transient lower respiratory tract infection involving mild to moderate pneumonia, therefore not mimicking severe human disease associated with MERS-CoV. However, these studies match observations from the lone autopsy report that MERS-CoV targets primarily alveolar pneumocytes. They conflict on whether lung endothelial cells are infected, which has been observed in cell culture and could facilitate systemic dissemination by allowing viral escape from the lungs ^33^.

### Common marmoset model

Efforts by several groups to develop a non-human primate model of severe MERS-CoV–induced disease have used common marmosets. Falzarano *et al*. ^34^ first reported that infection of marmosets with MERS-CoV causes severe pneumonia, lethal in some subjects, that appears to recapitulate human disease. Both this group ^35^ and Yu *et al*. ^36^ have subsequently compared MERS-CoV infection of marmosets with that of rhesus macaques and observed more severe disease, more robust viral replication, and more severe pathology and inflammatory cell lung infiltration in marmosets than in rhesus macaques. These reports indicate that marmosets can serve as a suitable model for severe human disease caused by MERS-CoV and support a role for immune-mediated pathology in the lungs as a factor in severe disease.

However, confounding these results, Johnson *et al*. ^37^ reported that intratracheal infection of marmosets with MERS-CoV results in only mild pneumonia and minimal viral replication in the lungs. They found no significant differences in disease between MERS-CoV–infected marmosets and marmosets inoculated with inactivated MERS-CoV, suggesting that the volume of the intratracheal viral inoculum itself might result in airway pathology. However, Yu *et al*. infected solely by the intratracheal route, rather than through multiple routes as Falzarano *et al.* did, and inoculated the marmosets with 10-fold less virus than Johnson *et al*. did. Like Johnson *et al.*, Yu *et al.* compared pathology in MERS-CoV infected marmosets and mock-infected marmosets, seemingly confirming the original finding by Falzarano *et al.* that viral infection induces the observed severe pathology in MERS-CoV infected marmosets. Resolving the differences between these studies will require considerably more work, but in the interim, the common marmoset appears to be a useful model organism for studying the pathogenesis of severe MERS-CoV disease, while the rhesus macaque may be appropriate for studying milder, likely under-reported human disease as well as for vaccine studies.

## Mouse models of MERS-CoV

MERS-CoV does not replicate in mice, because mouse DPP4 (mDPP4) does not support MERS-CoV entry ^38^, and this is due to two amino acid differences relative to human DPP4 (DPP4) in the region that interacts with Spike ^39^. Therefore, the development of mouse models has largely involved replacement of mouse *Dpp4* with human *DPP4* or modification of mDPP4 to render it compatible with Spike ^40^. The first mouse model established, before the generation of transgenic mice, used adenovirus-mediated transient expression of hDPP4 in the mouse airway via adenovirus-vectored transduction. Zhao *et al*. ^41^ reported that transient hDPP4 expression rendered mice susceptible to MERS-CoV replication in the lungs and the development of signs and symptoms of pneumonia. However, mice recovered and cleared the virus by 8 days post-infection, failing to recapitulate severe human disease.

Subsequently, several models using transgenic human *DPP4*-expressing mice were developed yet suffered from significant limitations in their ability to recapitulate human disease. The first transgenic mouse model was described by Agrawal *et al*. ^42^ in 2015 and further characterized in 2016 ^43^ and used mice globally expressing hDPP4. These mice do develop pneumonia as seen in humans, but the virus disseminates systemically, including robust viral replication in the brain. Also in 2015, Zhao *et al*. ^44^ described a similar transgenic mouse model, which likely has limited utility for pathogenesis studies due to systemic dissemination and severe neurological disease, but is suitable for studying the efficacy of vaccines and antiviral drugs. Similar results were reported in 2016 by Li *et al*. ^45^, although this group also used hDPP4 expressed under control of a lung-specific promoter, observing no disease following MERS-CoV infection.

More recently, three models using transgenic mice that appear to better recapitulate severe human disease have been published. The first of these, described in 2015 by Pascal *et al*. ^46^ and further detailed in 2017 by Coleman *et al*. ^47^, uses mice with the full-length mouse *Dpp4* gene replaced by its human equivalent. hDPP4 tissue distribution and expression levels in this system were largely equivalent to that of mDPP4 in wild-type mice. These mice support robust MERS-CoV replication in the lungs with little or no dissemination of the virus to other organs. All mice infected with the highest dose tested—2.5 × 10 ^4^ plaque-forming units (pfu)—developed severe lung pathology, lost 20% of body weight by day 7 post-infection, and were euthanized. Notably, and unlike in models described below, these mice succumb to infection with wild-type, rather than mouse-adapted, MERS-CoV. This may make it particularly useful for extension of *in vitro* studies of mutations found in clinical isolates such as a 48-nt deletion in ORF4a ^48^ or of mutations engineered into other viral proteins intended to disrupt their interactions with host immune responses.

Two mouse models, published in late 2016 and early 2017, use mice with *Dpp4* modified using CRISPR/Cas9 to serve as a functional receptor for MERS-CoV. Cockrell *et al*. ^40^ made two amino acid substitutions in mDPP4 previously determined to enable its usage by MERS-CoV ^39^, at positions 288 and 330. These mice support robust replication in the lungs following intranasal inoculation with 5 × 10 ^5^ pfu of wild-type MERS-CoV or MERS-CoV adapted on mouse NIH-3T3 cells expressing the chimeric mDPP4 with substitutions of amino acids 288 and 330. However, these mice exhibited no clinical signs of disease and minimal lung pathology. Despite the robust replication, 15 serial passages of virus were required to achieve lethality, significant declines in respiratory function, and severe lung pathology. The mouse-adapted virus contained several mutations, as expected, as well as a large deletion in ORF4b. This deletion may suggest that this protein is non-essential in the mouse, or its loss may represent an adaptation to virulence possibly because loss of this putative interferon (IFN) antagonist results in enhanced immune-mediated pathology.

Li *et al*. ^49^ developed a similar model in which they replaced exons 10–12 of mouse *Dpp4* with their human equivalents, rendering mDPP4 a functional receptor for MERS-CoV. As with the model developed by Cockrell *et al*., wild-type MERS-CoV replicated robustly in the lungs of these mice but did not cause disease. Virus serially passaged 31 times was lethal to 80% of mice infected with 2 × 10 ^6^ pfu. Notably, and supporting the emerging picture of immune-mediated pathology playing a significant role in disease, mouse-adapted MERS-CoV used in this model induced significantly more robust activation of innate immune and inflammatory genes than wild-type MERS-CoV and increased infiltration of the airway by innate immune cells.

## Innate immune suppression

Like many viruses, coronaviruses encode proteins to enable evasion or suppression of the host innate immune response, particularly that driven by the expression of antiviral type I and type III IFNs. Notably, however, the IFN response to coronavirus infection occurs remarkably late compared with many other viruses. MERS-CoV induces little detectable IFN or IFN-stimulated gene (ISG) expression early in infection of primary airway epithelial cells ^50^, *ex vivo* lung cultures ^51^, or immortalized airway-derived epithelial cells ^52^. In Huh7 hepatoma cells, no type I IFN transcript could be detected even 48 hours post-infection (hpi) ^53^, although Menachery *et al*. ^54^ have shown that Calu-3 airway-derived cells do mount an IFN response by 24 hpi with a high concentration of MERS-CoV.

Accordingly, considerable research has been conducted to identify the viral proteins which so dramatically delay the host immune response. Of particular interest are the lineage-specific accessory proteins (NS3, NS4a, NS4b, NS5, and NS8b) encoded in the 3′ end of the MERS-CoV genome. Conserved MERS-CoV proteins such as nsp1 ^55^, the nsp3 papain-like protease domain ^56, 57^, and the structural M protein ^58, 59^ may also counteract the host immune response but likely do so by similar mechanisms as their closely related orthologs in other coronaviruses. The lineage-specific accessory proteins, in contrast, lack homology to known viral or host proteins, making them of particular interest as their mechanisms of action may be unique among coronaviruses. Notably, NS4a and NS4b have been demonstrated by multiple groups to have IFN antagonism capabilities, and NS5 may as well ^58^. However, few of these studies have investigated the role of these proteins during infection. Instead, most studies have used ectopically expressed viral proteins, an experimental approach which offers the advantage of avoiding the need for high containment and isolation of a protein’s activity from those of other viral proteins.

The accessory proteins NS4a and NS4b, translated from the same bicistronic mRNA4, have also been identified as innate immune antagonists. NS4a was rapidly identified as a double-stranded RNA (dsRNA) binding protein which, though unique to MERS-CoV and MERS-like coronaviruses, has an RNA binding domain homologous to that of several cellular proteins ^60^. NS4a antagonism of IFN gene expression has been demonstrated by three research groups using very similar luciferase reporter assays ^58, 60, 61^ but, like M, not during actual virus infection. Notably, Niemeyer *et al*. ^61^ showed that NS4a binds the dsRNA mimic polyI:C and co-localizes with dsRNA during MERS-CoV infection, whereas Siu *et al*. ^60^ demonstrated that IFN antagonism is dependent on dsRNA binding. More recently, in 2016, Rabouw *et al*. ^53^ reported that ectopically expressed NS4a inhibits activation of the antiviral dsRNA binding protein PKR, preventing translation arrest and stress granule formation. However, infection with recombinant MERS-CoV∆ORF4 did not induce stress granule formation, and whether this virus activated PKR was not reported, leaving open the question of whether NS4a functions as a PKR antagonist during MERS-CoV infection.

NS4b was initially identified as a putative IFN antagonist by Yang *et al*. ^58^ in 2013 and Matthews *et al*. ^62^ in 2014, using luciferase reporter assays for IFNβ gene expression, and Yang *et al*. showed that NS4b—along with NS4a, M, and NS5—inhibited nuclear translocation of IRF3. Notably, Matthews *et al*. found that, although NS4b localizes primarily to the nucleus, deletion of the N-terminal nuclear localization sequence did not abrogate NS4b inhibition of IFNβ promoter-driven luciferase expression, a result confirmed by Yang *et al*. ^63^ in 2015. In that 2015 study, Yang *et al*. expanded on their earlier work, showing that ectopically expressed NS4b inhibited IFNβ promoter-driven luciferase expression as a consequence of overexpression of the IFN activators MDA5, RIG-I, MAVS, IKKε, TBK1, IRF3, and IRF7. MDA5 and RIG-I are cellular sensors of viral dsRNA, whereas MAVS transduces their recognition of dsRNA through the kinases IKKε and TBK1 to phosphorylate IRF3/7 and induce IFN. They demonstrate that NS4b associates with several of these antiviral proteins, preventing the formation of an IKKε/MAVS complex required to induce IFN, yet the authors identify, but do not characterize, an IFN antagonist function mediated specifically in the nucleus. Finally, in 2016 ^64^, we identified NS4b as a 2′,5′ phosphodiesterase (PDE) with structural homology to the mouse hepatitis virus (MHV) NS2 protein as well as a larger host protein family known as 2H-phosphoesterases. We previously characterized MHV NS2 as an RNase L antagonist ^65^ and showed that NS4b can functionally replace MHV NS2. Additionally, we used recombinant live MERS-CoV to demonstrate that a mutation in an NS4b catalytic residue (H182R) that abrogates its enzymatic activity results in RNase L activation in Calu-3 cells late during infection.

## Conclusions

Collectively, a great deal of progress has been made in this areas over the last several years, but it is also clear that the field of MERS-CoV research is still in its infancy. With respect to the origins of MERS-CoV, these are increasingly understood, yet it remains true that no virus directly ancestral to MERS-CoV, or containing a Spike S1 subunit with high similarity to MERS-CoV, has been either isolated or identified by sequence. Additionally, while the host switch from bats to dromedary camels likely occurred in Africa, banked Saudi Arabian camel serum dating to 1993 is seropositive for MERS-CoV ^66^, suggesting that the virus was also circulating there over 20 years ago, as in Africa.

Recent experimental work in camels supports their status as a MERS-CoV reservoir. A hallmark feature of a virus-reservoir host interaction is that for the virus to be maintained in a reservoir host population it should cause minimal to no disease in that host. Experimental infection of Jamaican fruit bats by Munster *et al*. ^67^ resulted in replication and shedding without clinical disease, although the ability to extrapolate from these conclusions is limited as these bats are not a putative MERS-CoV reservoir. More notably, and in support of the identification of dromedary camels as a reservoir seeding human infections, MERS-CoV infection of camels in natural and experimental settings results in either subclinical or causes only mild, transient upper respiratory tract disease ^68– 70^.

Further work to better understand the ecology of MERS-CoV should include continued intensive serosurveys, efforts to isolate bat coronaviruses, and acquisition and analysis of additional camel-derived MERS-CoV sequences to date and geolocate MERS-CoV evolution and host-switching. Such work will further our understanding of processes underlying zoonotic emergence of novel viruses, an ever-growing threat in a changing climate.

With respect to animal models, useful non-human primate and mouse models have been developed, yet work remains to reconcile the differences between them. Rhesus macaques may be more readily available than marmosets yet, because they develop only mild disease, may be less useful for studies of pathogenesis and therapeutics. Additionally, strikingly conflicting results of marmoset MERS-CoV infection, depending on the research group, require further reconciliation before this model should be widely adopted.

The development of MERS-CoV mouse models is an impressive scientific achievement, but the differences in infection outcome between the mice expressing hDPP4 in the mDPP4 locus and the mice expressing chimeric DPP4s require further study. Notably, the latter two models required mouse adaptation of the virus and still require 10- to 100-fold more virus to achieve severe disease. It is possible that full replacement of mDPP4 disrupts its non-MERS-CoV–related functions, including T-cell homeostasis, that could affect the course of disease, and the use of different MERS-CoV strains may also affect outcome. A side-by-side comparison of these models will help in elucidating and reconciling these differences. With respect to pathogenesis, all three models appear to recapitulate the pneumonia observed in humans, but the model using wild-type virus may offer certain advantages. Particularly, it will better allow studies of naturally occurring and engineered mutations in viral proteins, on the genetic background of human isolates, particularly the accessory proteins in the 3′ end of the genome. Several of these proteins have been identified *in vitro* as putative innate immune antagonists, and the potential to extend these studies to characterizing the role of these proteins in pathogenesis offers exciting opportunities for the field.

The area of what unique mechanisms MERS-CoV uses to evade and counteract the host immune response remains ripe for further study. None of the MERS-CoV accessory proteins shares significant amino acid identity or similarity with known coronavirus proteins, suggesting that their interactions with the host may be unique to the lineage C betacoronaviruses. In contrast, replicase and structural genes that interfere with the immune response do remain of interest but, owing to their conservation among coronaviruses, are less likely to act in unique or newly recognized ways. Although NS4a, NS4b, and NS5 have been reported to interfere with the innate immune response, most of this work, including that related to IFN induction, has been done using ectopically expressed protein and reporter assays. Exclusive use of ectopically expressed protein raises concerns about whether results may be skewed by non-physiological expression levels, mislocalization, or loss of possible interactions with other viral proteins. Although studies using live virus are more cumbersome and require biosafety level-3 containment, they are essential and feasible given the existence of multiple systems for generating recombinant MERS-CoV.
